# Cobalamin (Vitamin B12) in Anticancer Photodynamic Therapy with Zn(II) Phthalocyanines

**DOI:** 10.3390/ijms24054400

**Published:** 2023-02-23

**Authors:** Vanya Mantareva, Ivan Iliev, Inna Sulikovska, Mahmut Durmuş, Ivan Angelov

**Affiliations:** 1 Institute of Organic Chemistry with Centre of Phytochemistry, Bulgarian Academy of Sciences, Bld. 9, 1113 Sofia, Bulgaria; 2 Institute of Experimental Morphology, Pathology and Anthropology with Museum, Bulgarian Academy of Sciences, Bld. 25, 1113 Sofia, Bulgaria; 3 Department of Chemistry, Gebze Technical University, Gebze 41400, Turkey

**Keywords:** phthalocyanines, cobalamin, vitamin B12, photodynamic therapy (PDT), breast cancer cells, epithelial and fibroblast cell lines

## Abstract

Photodynamic therapy (PDT) is a curative method, firstly developed for cancer therapy with fast response after treatment and minimum side effects. Two zinc(II) phthalocyanines (3ZnPc and 4ZnPc) and a hydroxycobalamin (Cbl) were investigated on two breast cancer cell lines (MDA-MB-231 and MCF-7) in comparison to normal cell lines (MCF-10 and BALB 3T3). The novelty of this study is a complex of non-peripherally methylpyridiloxy substituted Zn(II) phthalocyanine (3ZnPc) and the evaluation of the effects on different cell lines due to the addition of second porphyrinoid such as Cbl. The results showed the complete photocytotoxicity of both ZnPc-complexes at lower concentrations (<0.1 μM) for 3ZnPc. The addition of Cbl caused a higher phototoxicity of 3ZnPc at one order lower concentrations (<0.01 μM) with a diminishment of the dark toxicity. Moreover, it was determined that an increase of the selectivity index of 3ZnPc, from 0.66 (MCF-7) and 0.89 (MDA-MB-231) to 1.56 and 2.31, occurred by the addition of Cbl upon exposure with a LED 660 nm (50 J/cm^2^). The study suggested that the addition of Cbl can minimize the dark toxicity and improve the efficiency of the phthalocyanines for anticancer PDT applications.

## 1. Introduction

Photodynamic therapy (PDT) has been well accepted as an emergency curative method with prompt therapeutic results and as an appropriate approach to diminish the development of drug resistance of tumor cells [1,2]. Phthalocyanine complexes (MPcs) have been recognized as promising PDT agents with several advantages over the firstly clinically approved porphyrin derivatives [3,4,5]. Previous studies showed that some tetra- substituted cationic MPcs have much higher efficacy than the related octa-substituted MPcs analogues [6]. This could be due to the larger size of the molecules of the octa-substituted MPcs, but also due to the binding to the lipopolysaccharides (LPS), which could prevent MPcs cellular internalization [7]. The comparison study of MPcs with the different position of the methylpyridinium-4-yl moieties suggest that the non-peripheral MPcs are more effective for PDT due to advanced photophysical parameters, while the peripheral MPcs show only a modest reduction in survival [8]. Zinc(II) phthalocyanines (ZnPcs) have been reported with relatively high PDT activity on tumor cells [9]. The mechanism of photosensitization (Type II) with the participation of phthalocyanines involves the singlet oxygen generation [10]. Type II photosensitization includes the production of triplet state molecules (^3^PS*) which react or annihilate themselves by an energy transfer with the formation of highly reactive singlet oxygen (^1^O_2_*), which is harmful to the tumor cells.

A previous study reported that the tumor cellular resistance can occur due to PDT upon treatment with numerous photosensitizers on different tumor models [11]. Previous studies showed that PDT along with sulfonated Zn(II)-phthalocyanine (ZnPcS_4_) resulted in the resistant tumor cells after many sets of PDT treatments but without necessary viability of the cells [12]. It was observed that the formation of viable MCF-7 cells due to PDT was the result of the increased p-glycoprotein expression which may prevent the further cellular uptake [13]. The observations about the resistance of cancer cells suggested that it is an interning property of the primary tumor that drains by various phenotypic and genotypic alterations [14].

Cobalamins (vitamin B12) were isolated and their complex structure was clarified after more than a century of research and developments on these compounds [15,16]. Chemically, four cobalt corrin complexes are known with structures that have different one axial substitution group, namely cyano (CN), hydroxyl (OH), methyl (CH_3_), or 50-desoxyadenosyl (Ado), and a second axial group of 5,6-dimethylbenzimidazole. There are two known synthetic forms, such as cyanocobalamin (CNCbl) and hydroxycobalamin (OHCbl), and two natural derivatives, such as methylcobalamin (MeCbl) and 50-deoxyadenosylcobalamin (AdoCbl). The typical very low photostability was determined for MeCbl and AdoCbl, with a tendency to produce the stable OHCbl at room temperature and in aqueous solution [17]. 

The therapeutic role of cobalamins (vitamin B12) in cancer treatment is attributed to an increase in the effectiveness of other antitumor agents [18,19,20,21,22]. However, there are no previous studies on phthalocyanines and cobalamin for PDT applications. A recent study reported the property of B12 to improve porphyrin delivery and cellular accumulation because of an uptake pathway via a receptor typical for cobalamin [19]. This study also reported the cell surface molecule (CD320) which can facilitate the porphyrins entry in cancer cells. Moreover, considering that Cbl as an essential co-factor for DNA synthesis, the surface expression of CD320 increases upon proliferation. This could contribute to the accumulation of porphyrins in the tumor cells and can be a useful approach for the enhancement of drug selectivity. The recent studies demonstrated the chemically functionalized hybrid such as vitamin B12 on a microalgae’s surface and a photoactive rhenium(I) tricarbonyl anticancer complex with enhanced anticancer activity [20,21]. The targeted delivery to cancer cells, while leaving the healthy tissue undamaged, can reduce general toxicity of anticancer drugs [23]. Most studies with vitamin B12 have proven that, when it is used in combined therapy, it contributes to increasing the effectiveness of anticancer drugs [24]. Moreover, the deficiency of B12 is known to correlate with increased micronucleus formation and carcinogenesis. Additionally, it was shown that B12 contributes to a distinctive mechanism of cell death known as paraptosis-like cell death. 

**Figure 1:**
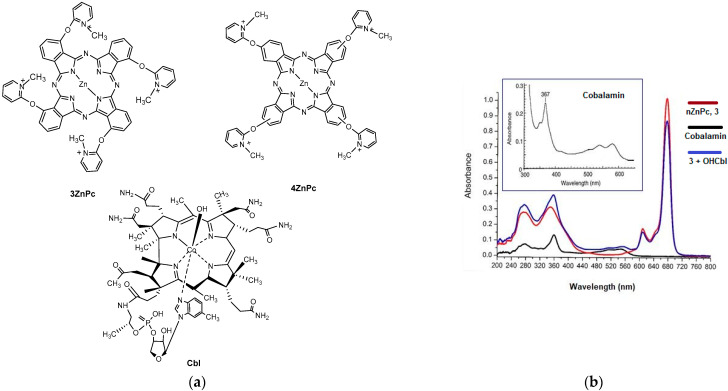
Structures of the studied two Zn(II)-phthalocyanines (3ZnPc and 4ZnPc) and hydroxycobalamin (Cbl) (**a**). The absorption spectra of compounds in a concentration of 10^−5^ M in DMSO (the spectrum of Cbl on top is magnified) (**b**).

The current study aims to investigate the anticancer photodynamic potential of two tetra- methylpyridiloxy substituted Zn(II)-phthalocyanines, which are differing in the position of the substituents, namely the non-peripheral for 3ZnPc and the peripheral for 4ZnPc complex (Figure 1). Both zinc(II) phthalocyanines (3ZnPc and 4ZnPc) were evaluated as photosensitizers for PDT on two breast cancer cell lines (MCF-7 and MDA-MB-231) and a non-tumorigenic cell line (MCF-10A) as the normal breast cells. The efficacy and selectivity of 3ZnPc was examined in comparison to the previously studied 4ZnPc [25,26]. The cytotoxicity for the mixture with Cbl was evaluated for the more toxic in the dark condition of 3ZnPc complex. The photo-safety tests were carried out on an embryonal mouse fibroblast cell line (BALB/c 3T3) as a well-known model cell line with higher sensitivity than adult cells upon investigation of the new compounds. The soft irradiation (10 J/cm^2^) was applied using a LED *Helios-iO* solar simulator with a wide spectrum (360–960 nm). The specific irradiation was used in red visible spectrum from a LED 660 nm (50 J/cm^2^ and 100 mW/cm^2^) for PDT studies with phthalocyanines.

## 2. Results

**Scheme 1:**
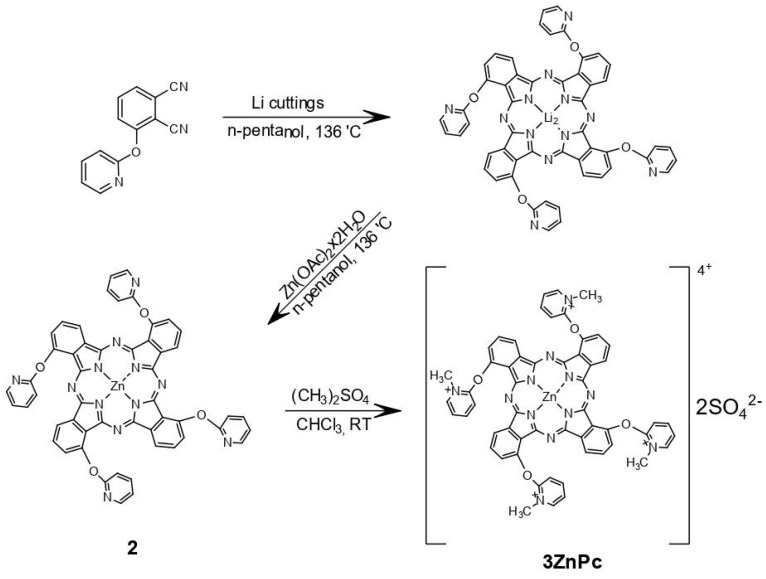
Synthesis of non-peripherally substituted Zn(II)-phthalocyanine (3ZnPc).

The synthesis of two tetra-methylpyridiloxy substituted Zn(II)-phthalocyanines (3ZnPc and 4ZnPc) which differ in the position of the substitution groups were carried out according to a recently published synthetic procedure [27,28]. The synthesis of non-peripherally methylpyridiloxy substituted Zn(II)-phthalocyanines (3ZnPc) is shown in Scheme 1. The cyclotetramerization included the preparation of lithium phthalocyanine (Li_2_Pc) which further continued with a metal insertion. This step permits to shorten the reaction time. The next step of an addition of zinc salt was applied in order to obtain the zinc complexes. This pathway permits to use Li_2_Pc as a starting compound in the further reactions of synthesis of different metal phthalocyanine complexes. The quaternization reaction was done with dimethylsulphate to obtain the final water-soluble compounds with non-peripheral substitution groups to macrocycle. 

A hydroxycobalamin (Cbl) was used after an additional purification step of the commercial product for the study of absorption spectra and photobiology of the photodynamic activity of both ZnPcs. The Cbl compound was also studied for the photo-safety on normal cells and for the cytotoxicity study on both breast cancer model cell lines by an application of the light source with a solar light spectrum of irradiation. Considering the complex ring structure of Cbl, the compound was also evaluated as a photosensitizer for photodynamic therapy (PDT). 

The absorption spectra of 3ZnPc and Cbl are presented in Figure 1. As can be seen, the absorption maxima of the single compound 3ZnPc and of the mixture with Cbl compounds (3ZnPc and Cbl) are overlapped in the UV region with an increment of optical density because of the absorption of Cbl. Both spectra were recorded in biocompatible solvents such as dimethylsulfoxide (DMSO) for concentrations of 10^−5^ M.

**Figure 2:**
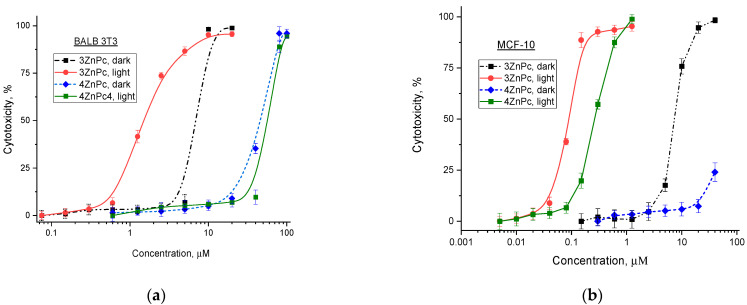
Dark and photo-cytotoxicity of zinc(II) phthalocyanines with non-peripheral groups for 3ZnPc and peripheral groups for 4ZnPc studied on two normal cell lines: (**a**) normal BALB 3T3 cell line and (**b**) non-tumorigenic MCF-10A cell line.

The dark and photocytotoxicity of the complexes 3ZnPc and 4ZnPc were studied on a normal cell line BALB 3T3 (Figure 2a). The results on normal cells BALB 3T3 showed a lower photo-safety (high phototoxicity) for the non-peripherally substituted 3ZnPc as compared to the peripherally substituted 4ZnPc. There is a slight difference after irradiation of the normal cells with 4ZnPc which suggests the lack of phototoxicity of peripherally substituted 4ZnPc. The results for the normal cell line BALB 3T3 showed a similar behavior with a huge concentration gap between the samples with and without irradiation. As can be seen, a higher phototoxicity was observed for 3ZnPc by irradiation at very low concentrations (<0.1 µM) and a significant dark toxicity for a concentration of ~100 µM (Figure 2a). 

The phototoxicity of 4ZnPc was observed for concentrations between 0.1–1 µM and almost without dark toxicity on a non-tumorigenic cell line MCF-10A (Figure 2b). The results were observed upon irradiation with a specific for PDT light emission diode with a maximum at 660 nm (LED 660 nm) specific for PDT and the properly selected parameters (60 mW/cm^2^ and 50 J/cm^2^). The non-peripherally substituted 3ZnPc was evaluated for both cell lines with the high phototoxic effect for low concentrations around 0.1 µM (>50% cell viability). The studies on tumor cell lines with 3ZnPc and 4ZnPc showed the phototherapeutic index (PI) for both the tumor cell lines (MCF-7 and MDA-MB-231); the index PI was 180 and 162, respectively, for peripheral 4ZnPc, while 112 for MCF-10A. The values of PI for tumor cells were significantly higher as compared to the values for the normal cell line, which suggested the high impact of phthalocyanine compounds as a photoactive agent. The relatively high phototoxic effect was observed on MCF-10A treated with 4ZnPc (PI = 294). The studies showed very low photo-safety and a high photocytotoxicity for non-peripheral 3ZnPc, as well as reduced toxic action by one order for peripheral 4ZnPc. This means that for the cells located in the light spot after proper irradiation, the normal tissues can also be damaged to a higher extent by 3ZnPc. Furthermore, the observed phodynamic effect of 4ZnPc was determined as relatively weak for more aggressive tumor cell line MDA-MB-231 (PI = 53). Though, the significant dark toxicity of 3ZnPc is a limitation for PDT applications. 

**Figure 3:**
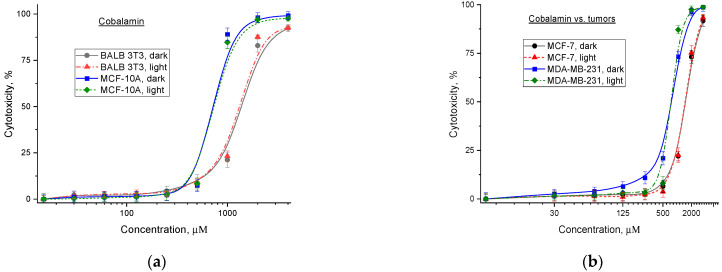
Dark and photo-cytotoxicity curves of cobalamin (Cbl) studied on: (**a**) normal BALB 3T3 cell line and non-tumorigenic MCF-10A cell lines; and (**b**) MCF-7 and MDA-MB-231 tumor cell lines.

Cobalamin (vit. B12) was evaluated to have low cytotoxicity independent of the applied light irradiation. The obtained results for the non-tumorigenic MCF-10A cells, as well as for the normal cell line BALB 3T3, in the dark and after irradiation, showed similar curves (Figure 3a). The cytotoxic effect of Cbl on both tumor cell lines (MCF-7 and MDA-MB-231) was determined in a dose-increasing condition with a difference between the sigmoidal curves, which was not significant considering the photo- and dark cytotoxicity. The calculated mean CC_50_ values (0.8–1.5 µM) indicated very low levels of cytotoxicity for this porphyrin-like compound. The tested tumor and normal cells were determined as typical for non-phototoxic compounds PIF~1. Moreover, up to a concentration of 200 µM Cbl, there was a minimal difference of *p* < 0.001 as compared to the negative control. This suggests that Cbl has minimal cytotoxicity as well as on the studied tumor cell lines independent of the light exposure for the applied wide concentration range. Similar results were obtained for both the tested tumor cell lines incubated with Cbl for the concentrations >5 mM, which suggested the selectivity behavior of Cbl (Figure 3b). The lack of difference in the toxicity of Cbl suggested the non-photodynamic mechanism of action on tumor cells.

**Figure 4:**
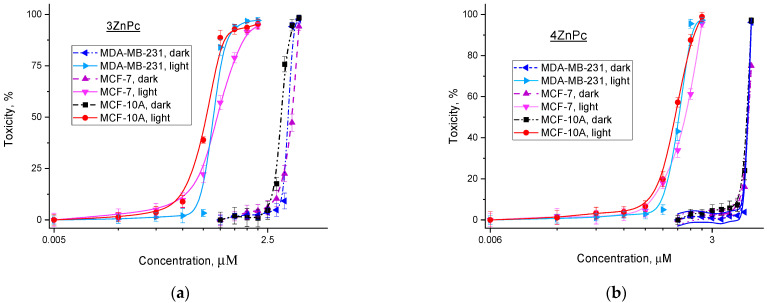
Dark and photo-cytotoxicity of zinc(II) phthalocyanines differing in the position of substitution groups ((3ZnPc and 4ZnPc) presented as followed: (**a**) 3ZnPc and (**b**) 4ZnPc on two breast tumor cell lines (MCF-7 and MDA-MB-231) and a non-tumorigenic MCF-10A cell line.

Non-peripherally substituted 3ZnPc was evaluated as a more powerful photosensitizer in comparison to peripherally substituted 4ZnPc for the tested tumor cell lines. The presented curves for 3ZnPc on tumor cell and the non-tumorigenic cells showed a concentration gap between the samples with and without irradiation for both of the tested ZnPcs (Figure 4).

**Figure 5:**
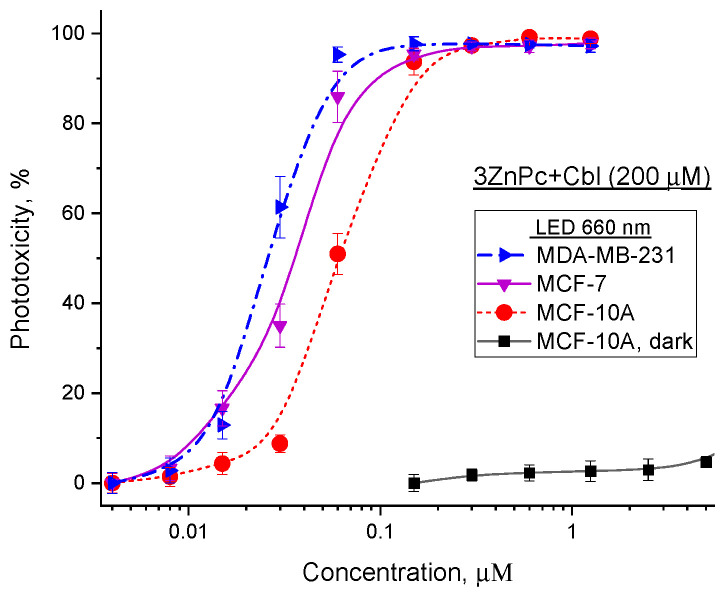
Photocytotoxicity of non-peripherally substituted zinc(II) phthalocyanine (3ZnPc) by addition of 200 µM cobalamin (Cbl) on breast cancer cell lines MCF-7 and MDA-MB-231 in comparison to a non-tumorigenic MCF-10A cell line.

The addition of a second compound, such as Cbl, showed a tendency to minimize the high dark toxicity of 3ZnPc due to lower concentrations for the similar efficiency (Figure 5). An improvement to the selectivity between tumor normal cells was observed for the mixture of 3ZnPc and Cbl. As can be seen, 3ZnPc showed complete cell death for concentrations <0.1 µM at the irradiation only with LED 660 nm (100 mW/cm^2^ and 50 J/cm^2^). The calculated results for 3ZnPc are summarized in Table 1. They showed that the addition of a biologically active compound, such as Cbl, increased the photodynamic effect (PI) of 3ZnPc for lower concentrations. Moreover, the highest efficiency of the combined application of compounds Cbl and 3ZnPc was observed for the more aggressive MDA-MB-231 cells (PI = 500). In addition, the mixture of 3ZnPc + Cbl was shown to lead to a high selectivity of the photocytotoxicity tumor vs. normal cells (SI = 2.31). The results of Cbl to 4ZnPc suggested that Cbl was not advanced by the cytotoxicity; moreover, the phototoxicity on normal cells was overlapped with the obtained for the tumor cell lines.

**Figure 6:**
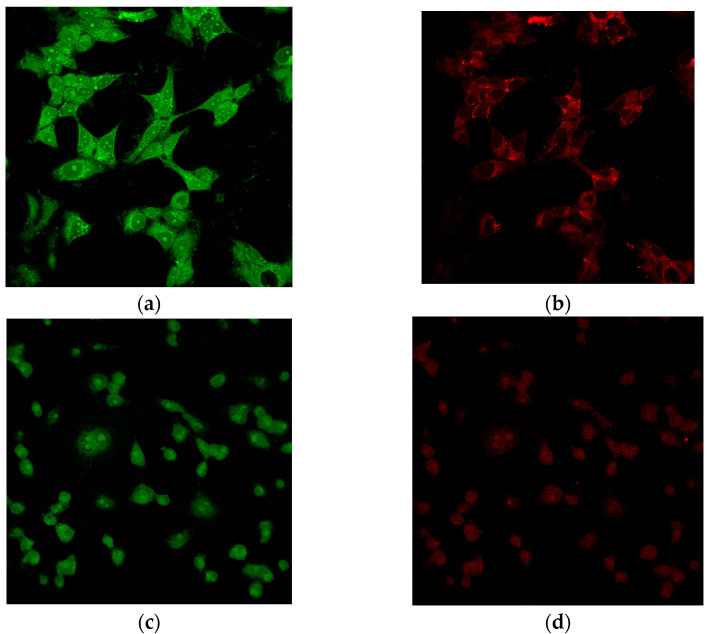
Confocal laser scanning microscope images of tumor cells with 3ZnPc: (**a**) MCF-7 cells (scale: exc: 405 nm; em: 450–550 nm); (**b**) incubated 3ZnPc (scale: exc: 635 nm; em: 670–780 nm); (**c**) MDA-MB-231 cells; and (**d**) 3ZnPc studied at the spectral conditions as (**a**,**b**) × 63.

Figure 6 shows the images obtained by the means of a confocal laser scanning microscope (CLSM). The study showed the accumulation of 3ZnPc in cytoplasmic reticulum. Upon irradiation with LED 660 nm, the re-localization of the compound was observed. As can be seen, the red signals are not very intensive because of the fluorescence spectrum in the region 670–780 nm. Both ZnPcs showed no significant difference in the localization, re-localization, and the fluorescence intensity of the signals.

## 3. Discussion

The present study showed the impact of cobalamin (Cbl) which is a popular vitamin (B12) on antitumor efficacy of two Zn(II)-phthalocyanines with methylpyridiloxy substitution groups on non-peripheral (3ZnPc) and peripheral positions (4ZnPc). The stable form hydroxycobalamin (Cbl) was used in the study due to the biological and photo-activity of this porphyrinoid-like compound (Figure 1). A high phototoxic action was observed even at low concentrations of 3ZnPc and 4ZnPc on both BALB 3T3 and MCF-10 as normal cell lines (Figure 2). The curves for dark toxicity showed the higher toxicity for 3ZnPc in comparison to 4ZnPc. The used Cbl was evaluated with high photo-safety on four tested cell lines (Figure 3). The antitumor activity of both complexes was tested on two breast cancer cell lines, which suggested the high effect at a lower concentration of 3ZnPc (Figure 4). However, the dark toxicity was also high for the photosensitizers. The addition of Cbl was observed to lower the dark toxicity. The effects of selectivity and a high photocytotoxicity were evaluated for lower 3ZnPc concentrations. These observations can be explained with the receptor mediated uptake and tumor-specificity of cobalamins. This reduction of 3ZnPc concentration beneficially led to minimizing the dark cytotoxicity to the normal cells (Figure 5). These observations can be explained by the non-covalent electrostatic and π–π interaction between both macrocycles with an extra production of the triplet excited state molecules, and by the cellular receptor for Cbl on the surface of tumor cells. The studies with confocal fluorescence microscope showed the ZnPcs localization in the cytoplasmic organelles with slight re-localization due to the light application (Figure 6).

The previous studies with different peripherally substituted Zn(II)-phthalocyanines (ZnPcs) suggested their efficiency for photodynamic therapy (PDT) on breast cancer cells [25,29]. Nevertheless, while there have been clinically accepted phthalocyanines since the early years of this century, the studies of these dyes are very actual because of some undesirable side effects [30,31]. One possible approach to minimizing a limitation such as high toxicity, is by the addition of the cell specific and photoactivable complex ring compound. Cobalamins have been a well-accepted form of medication acting as cofactor for DNA synthesis. However, very recently, B12 was reintroduced as a tunable and light-responsive stand in for well-known therapeutic agents [32]. The resistance problem was shown also to occur in some extent in PDT procedure, but its development was observed to happen not so rapidly after 10 cycles of PDT procedure, and with not as much survived cells as in the case of the chemotherapies [33,34]. Both chosen cancer cell lines (MCF-7 and MDA-MB-231) were previously evaluated with resistance towards some chemotherapeutic drug, such as cisplatin and doxorubicin. The isolation and an initial characterization of human MCF-7 cells with resistance to PDT was recently reported [14]. In the experiment, 20 μM phthalocyanine (ZnPcS_4_) and irradiation with a specific laser (20 J/cm^2^) was used. The PDT protocol was repeated in order to observe so-called MCF-7/PDT cells. According to the results using ZnPcS4-PDT, the conditions must be rigorous, intense, and prolonged to obtain PDT-resistant cells with high cellular viability. This study suggested that after 10-times repetitive procedure, the generation of resistance to the PDT protocol can be observed [14]. The mechanism of cellular killing includes the ability to induce apoptosis in response to PDT [35]. A selective tumor progression led to some re-growth of the cells which suggested the resistance to the applied treatment regime. The earlier results with actual PDT photosensitizers porphyrins were similar, as for example, the PDT-resistant tumor cells were isolated after treatment with clinical drugs Photofrin and ALA [36]. Although numerous anticancer drugs for breast cancer treatment have been developed over the years, it remains a therapeutic challenge to overcome the resistance problem with new original attempts [37,38]. The cancer cells were reported to initiate the metastasis and to become resistant to certain drugs, as well as exhibit lesions of recurrence after surgery. PDT has an advantage of limited toxicity and minimal side-effects through a procedure, and it also has a fast response with low ability for the development of resistance.

## 4. Materials and Methods

### 4.1. Photoactive Compounds

Zn(II)-phthalocyanines with methylpyridiloxy substitution groups have been synthesized following a recent synthetic scheme [25,26]. The chemical and solvents were used after an additional purification step. The study presented a synthesis of a non-peripheral Zn(II)- phthalocyanine (3ZnPc). The used hydroxycobalamin (Cbl) was a product of Sigma-Aldrich purchased from FOT (Sofia, Bulgaria) in a seated vial. Before the study, an additional purification step of Cbl was applied. It was used as a Soxhlet extraction with several organic solvents with increasing polarity to remove the decomposed compound and some other by-products. The final washing was done with an excess of hot ethanol. The pure compound was collected by centrifugation. Then, it was dried at 80 °C in a glass oven under argon.

#### 4.1.1. 2,(3),9(10),16(17),2 3(24)-Tetrakis-[(2-pyridyloxy) Phthalocyaninato]zinc(II), (2)

A few cuttings of lithium were dissolved in 1-pentanol (3 mL) at 60 °C. Then, 2-pyridyloxyphthalonitrile (0.221 g, 1 mmol) was added to the mixture. The temperature was risen to 137 °C and the reaction was kept under argon. After 3 h, a zinc acetate dihydrate (0.252 g, 1.15 mmol) was added and the stirring continued for 3 h. Then the mixture was cooled and the solution was dropped in cold n-hexane to form a fine sediment. The green solid was collected by centrifugation with a replacement of solvent until its transparency. The crude product was purified by column chromatography (SiO_2_) using DCM-MeOH (9:1). Yield: 0.120 g (54%). IR [ν_max_/cm^−1^]: 3097 (Ar-CH), 1652 (C=C), 1568, 1523, 1510, 1389, 1327, 1287, 1258, 1132, 1112 (C-O-C), 1045, 805, 744. ^1^H-NMR (CDCl_3_): δ, ppm 7.68–7.24 (14H, m, Pc-H and Pyridyl-H), 7.08–6.41 (14H, m, Pc-H and Pyridyl-H). MALDI-TOF-MS *m/z*: Calc. 948.16 for C_52_H_28_N_12_O_4_Zn; Found [M]^+^ 948.16; [M + Na]^+^ 971.12

#### 4.1.2. 2,(3),9(10),16(17),13(24)-Tetrakis-{[(2-(N-methyl)pyridyloxy]phthalocyaninato} Zinc (II) Sulphate (3ZnPc)

Phthalocyanine 2 (100 mg, 0.1 mmol) was heated to 120 °C in freshly distilled DMF (0.5 mL) in a closed apparatus and dimethyl sulphate (0.2 mL) was added dropwise. The reaction mixture was stirred at 120 °C overnight. Then, it was cooled to room temperature and the product was poured in a hot acetone. The formed fine hail was collected by centrifugation. The green solid was washed with an order of solvents such as hot ethanol, ethyl acetate, THF, chloroform, n-hexane, and diethyl ether. The hygroscopic product was dried over phosphorous pentoxide at 80 °C. Yield: 0.080 g (67%). IR [ν_max_/cm^−1^]: 3076 (Ar-CH), 1635, 1572 (C=C), 1531, 1471, 1326, 1223 (S=O), 1178, 1106 (S=O), 1026 (C-O-C), 931, 835, 764, 661 (S-O). ^1^H-NMR (DMSO-*d_6_*): δ, ppm 7.99–6.82 (28H, m, Pc-H and Pyridyl-H), 4.39 (12H, m, CH_3_). UV/Vis (DMSO), λ_max_, nm (log ε): 318 (4.36), 623 (4.11), 662 (4.21). MALDI-TOF-MS *m/z*: Calc. 1201.53 for C_56_H_40_N_12_O_12_S_2_Zn; Found 301.383 [(M + 4)/4]^+^.

### 4.2. Light Sources

Light-emitting diode lamp Helios-iO (SERIC Ltd., Tokyo, Japan) was applied in this study as an irradiation source. The fluence rate was determined by measurements with a power meter PM 100D with a sensor S120VC (Thorlabs Inc., North Newton, KS, USA). The working capacity was considered the spectrum between 200–1100 nm, with power in the range 50 nW–50 mW. The linearity of the sensor is ±0.5% for the used spectral area (280–980 nm) with lower than ±5%. The diameter of the sensors’ aperture is 9.5 mm. The determination of the intensity of the radiation light was achieved for the distance of 25 cm. Nevertheless, the natural diffuse light in the experimental area was relatively low (≈1.16 W/m^2^); all measurements were conducted in the dark place. The second light source used for PDT experiments was a light-emitted diode LED 660-nm (ELO Ltd., Sofia, Bulgaria) with a power density of 100 mW/cm^2^ and a light dose 50 J/cm^2^ applied in the experiments with phthalocyanines.

### 4.3. Cells’ Cultures and Cultivation

The mice embryonal fibroblasts BALB/c 3T3, A31 (ATCC^®^ CCL-163TM); the non-tumorigenic cell line from epithelium of breast MCF-10A (ATCC^®^ CRL-10317™); the luminal adenocarcinoma from breast gland MCF-7 (ATCC^®^ HTB-22™), type A (ER+, PR+, HER2-), and the triple negative carcinoma of breast gland MDA-MB-231 (ATCC^®^ HTB-26™), (ER-, PR-, HER2-) were used. The cultivation of the adhered cell cultures was done in DMEM medium (4.5 g/L glucose), 10% fetal calf serum, 100 U/mL penicillin, and 0.1 mg/mL streptomycin in plastic dishes with a working area of 25 cm^2^ and 75 cm^2^. The cells were kept in logarithmic phase of grown at 37 °C in 5% CO_2_ atmosphere. The cells’ samples for in vitro tests were prepared from the cells in an exponential stage of grown-up. After trypsinization, the cells were brought to the required concentration so that the cell density in each of the 96-well plates was fixed to 1 × 10^4^ cells per well. The cultivation was performed for 24 h. Then, the cells were incubated with compounds.

### 4.4. Photodynamic and Cytotoxicity Studies

Neutral red uptake in vitro test (NRU-assay) was applied to register the results after treatments. This is a color method for the evaluation of the cells’ viability in vitro. As is known, the method is based on the ability of the alive cells to accept in their lysosomes the dye neutral red. The mouse embryonal fibroblast cells (BALB/c 3T3, clone A31) were cultivated in dishes with an area of 75 cm^2^ as monolayer cells’ cultures at the standard conditions. The cells with a density of 1 × 10^4^ cells in 100 μL culture medium per well in 96-well plates were cultivated. The cell incubation was performed for 24 h at standard conditions to achieve a good adhesion. Then, they were treated with the studied phthalocyanines (3ZnPc and 4ZnPc) and cobalamin (Cbl), following the double increase of the concentration. The study was carried out separately in two plates simultaneously. One of the tested plates was kept in the dark place in order to study dark toxicity of photoactive compounds. The second plate for phototoxicity study was irradiated with LED Helios-iO with a constant dose of 0.64 J/cm^2^. The culture medium containing neutral red was added during the incubation time of 24 h. The wells were washed 3 h later with PBS, pH 7.4, and a solution of ethanol/ acetic acid. Distilled water in the ratio 49/1/50 was added. The optical density was measured by using the TECAN microplate reader, λ = 570 nm. The cellular toxicity was calculated following the Equation (1):

Cytotoxicity (%) = (1 − (OD_570_ (treated sample)/OD_570_ (negative control)) × 100 (1)

### 4.5. Parameters Determined from Cytotoxicity Studies

The evaluation of SI value (SI = CC_50_ of non-malignant cell line/CC_50_ of tumor cells) for any compound is very crucial for determining whether further works can be continued. For evaluating any anticancer activity of a sample, its cytotoxicity against a non-malignant cell line must be determined in order to calculate the SI value [39,40]. Weerapreeyakul et al. [41] proposed a lower SI value (>3) for the classification of a prospective anticancer sample. Rashidi et al. [42] considered the SI values of more than 2 as high selectivity.

The calculation of the measures of photocytotoxicity was performed on the basis of the set of discrete concentration-response values having to be approximated by an appropriate continuous concentration-response curve. The fitting of the curve to the data is commonly performed by a non-linear regression method. To assess the influence of data variability on the fitted curve, a bootstrap procedure is recommended.

A photo-irritation factor (PIF) is calculated using the following Formula (2):

PIF = IC_50_ (−Irr)/IC_50_ (+Irr) (2)

where −Irr is the absence of light and +Irr is the presence of light.

Phototherapeutic indices (PI) are reported as the ratio of dark to light EC50 values and used as a measure of light-induced potency [43,44]. The ratio between the IC50 value (half maximal inhibitory concentration) of the resting and the IC50 value of the activated compound should be as high as possible [45]. Phototherapeutic index was defined as the dark EC_50_ value divided by the light EC_50_ value and calculated using the Equation (3).

PI = dark EC_50_/light EC_50_ (3)

### 4.6. Cellular Localization Study

The samples were prepared after seeding the cells with a density of 10^5^ cells/mL on Teflon-coated diagnostic slides (Menzel-Glaser, Braunschweig, Germany) with volume 25 μL per well. The cultivation was continued for 24 h and after washing the further incubation (1.5 h) with ZnPcs. The samples of cells were fixed in ice-cold acetone for 20 min, dried, cleared in glycerol phosphate-buffered saline (PBS) (9:1 *v*/*v*), and three identical samples on a slide were prepared. The fixed cells were placed by using an oil immersion on a confocal laser scanning microscopy (CLSM) Leica TCS SPE microscope (Leica Microsystems, Mannheim, Germany). The cells were visualized at an excitation wavelength of 405 nm and an emission spectrum of 450–550 nm for the native autofluorescence. The phthalocyanine dye was visualized at an excitation of 635 nm and an emission spectrum of 670–780 nm with an amplification of ×63 (NA = 1.23).

### 4.7. Statistics

The experiments were carried out in triplicate and the data are presented as a mean value ± standard deviation (SD) by the Student’s test and the difference between two means was compared by an unpaired Student’s test. The values of *p* < 0.05 were considered as significant.

## 5. Conclusions

Two Zn(II)-phthalocyanines which are differing in positions of methylpyridiloxy substituents (3ZnPc and 4ZnPc) were studied as photosensitizers on breast cancer cell lines (MCF-7 and MDA-MB-231). The results showed much higher photo- and cytotoxicity for non-peripherally substituted complex (3ZnPc) in comparison to peripheral 4ZnPc. Both compounds were evaluated with dark toxicity for the tested normal cell lines. A second porphyrinoid, namely hydroxycobalamin (Cbl), was evaluated with high photo-safety at a solar spectrum of irradiation with a LED simulator (10 J/cm^2^) for the spectral range 360–960 nm. The cellular viability was not affected by the light exposure of cobalamin. The addition of Cbl to 3ZnPc resulted in the optimal PDT efficiency at lower concentrations of 3ZnPc, which diminished the dark toxicity of this compound. In addition, the improved selectivity of the cytotoxic action tumor vs. normal cells was observed. As general, PDT with two biologically and photo-active compounds (phthalocyanine and cobalamin) could have a positive effect on the selectivity and the dark toxicity of phthalocyanine complexes which are of interest for PDT applications.

## Figures and Tables

**Table 1:** Mean values of CC_50_ ± SD (µM) for cell cultures treated with 3ZnPc and with two compounds (3ZnPc and Cbl).

Cell Lines	Cytotoxicity	Phototoxicity	PIF *, PI **	SI ***
	3ZnPc, CC_50_ ± SD (µM)			
BALB 3T3	7.36 ± 0.12	1.58 ± 0.11	4.66 *	-
MCF-10A	8.96 ± 0.26	0.08 ± 0.005	112	-
MCF-7	21.6 ± 1.53	0.12 ± 0.008	180	0.66
MDA-MB-231	14.59 ± 0.26	0.09 ± 0.004	162	0.89
	3ZnPc + Cbl 200 µM, CC_50_ ± SD (µM)			
MCF-10A	28.74 ± 0.55	0.065 ± 0.005	440	-
MCF-7	9.41 ± 0.97	0.042 ± 0.001	225	1.56
MDA-MB-231	14.18 ± 0.22	0.028 ± 0.002	500	2.31

* PIF–Photo-irritation factor; PIF < 2 not phototoxic; 2 ≤ PIF < 5 slight phototoxicity; PIF ≥ 5 phototoxic; ** PI–Phototherapeutic index; *** SI–Selectivity index.

## Data Availability

The data presented in this study are available on request from the corresponding author.
